# The effectiveness of trauma-focused psychotherapy for complex post-traumatic stress disorder: A retrospective study

**DOI:** 10.1192/j.eurpsy.2022.2346

**Published:** 2022-11-25

**Authors:** Eirini Melegkovits, Jocelyn Blumberg, Emily Dixon, Kimberley Ehntholt, Julia Gillard, Hamodi Kayal, Tim Kember, Livia Ottisova, Eileen Walsh, Maximillian Wood, Rafael Gafoor, Chris Brewin, Jo Billings, Mary Robertson, Michael Bloomfield

**Affiliations:** 1 Traumatic Stress Clinic, Division of Psychiatry, University College London, London, United Kingdom; 2 Traumatic Stress Clinic, St Pancras Hospital, Camden and Islington NHS Foundation Trust, London, United Kingdom; 3 Research Department of Primary Care and Population Health, UCL, Royal Free Hospital, London, United Kingdom; 4 Clinical, Educational and Health Psychology, University College London, London, United Kingdom; 5 Translational Psychiatry Research Group, Research Department of Mental Health Neuroscience, Division of Psychiatry, University College London, London, United Kingdom; 6 National Institute for Health Research University College London Hospitals Biomedical Research Centre, University College Hospital, London, United Kingdom; 7 National Hospital for Neurology and Neurosurgery, University College London Hospitals NHS Foundation Trust, London, United Kingdom

**Keywords:** Complex post-traumatic stress disorder, CPTSD, EMDR, ICD-11, trauma-focused CBT

## Abstract

**Objective:**

We retrospectively evaluated the effectiveness of trauma-focused psychotherapy (TF-P) versus stabilization and waiting in a civilian cohort of patients with an 11th version of the international classification of disease (ICD-11) diagnosis of complex post-traumatic stress disorder (CPTSD).

**Methods:**

We identified patients with CPTSD treated at a specialist trauma service over a 3-year period by triangulating evidence from self-report questionnaires, file review, and expert-clinician opinion. Patients completed a phase-based treatment: stabilization consisting of symptom management and establishing safety, followed by waiting for treatment (phase 1); individual TF-P in the form of trauma-focused cognitive behavioral therapy (TF-CBT), or eye movement desensitization and reprocessing (EMDR) or TF-CBT plus EMDR (phase 2). Our primary outcome was PTSD symptoms during phase 2 versus phase 1. Secondary outcomes included depressive symptoms, functional impairment, and a proxy CPTSD measure. Exploratory analysis compared outcomes between treatments. Adverse outcomes were recorded.

**Results:**

Fifty-nine patients were included. Compared to receiving only phase 1, patients completing TF-P showed statistically significant reductions in PTSD [*t*(58) = −3.99, *p* < 0.001], depressive symptoms [*t*(58) = −4.41, *p* < 0.001], functional impairment [*t*(58) = −2.26, *p* = 0.028], and proxy scores for CPTSD [*t*(58) = 4.69, *p* < 0.001]. There were no significant differences in outcomes between different treatments offered during phase 2. Baseline depressive symptoms were associated with higher PTSD symptoms and functional impairment.

**Conclusions:**

This study suggests that TF-P effectively improves symptoms of CPTSD. However, prospective research with validated measurements is necessary to evaluate current and new treatments and identify personal markers of treatment effectiveness for CPTSD.

## Introduction

The 11th version of the international classification of diseases (ICD-11) [1] introduced complex post-traumatic stress disorder (CPTSD). PTSD and CPTSD represent distinct diagnostic entities [1, 2]. CPTSD commonly arises following exposure to prolonged and repetitive interpersonal traumas, where escape is difficult or impossible [3]. These may include sexual, physical, and emotional abuse in childhood and adolescence, torture, genocide, prolonged domestic violence, and/or institutional abuse [4–6]. Compared to chronic PTSD, a CPTSD diagnosis requires disturbances of self-organization (DSO), namely emotional dysregulation; a negative self-concept; and impaired interpersonal relationships [1, 2] alongside core PTSD symptoms, that is, re-experiencing through flashbacks and intrusive memories, avoidance of trauma-related reminders, and heightened threat sensitivity. Early evidence suggests an impairment in the neural circuitry involved in threat processing [7] and response inhibition [8] in individuals with CPTSD, reflecting the additional emotion dysregulation, compared to those with PTSD without complex symptoms. Finally, patients with ICD-11 CPTSD show higher levels of suffering, comorbidity, and functional impairment than those with ICD-11 PTSD [9–15] and DSM-5 PTSD [16, 17].

International guidelines on CPTSD management [18, 19] recommend a phase-based psychotherapeutic approach [20, 21]. Meta-analyses also support the effectiveness of psychological interventions in patients with symptoms of CPTSD [22–24]. Trauma-focused cognitive behavioral therapy (TF-CBT) and eye movement desensitization and reprocessing (EMDR) have the strongest evidence base for core PTSD symptoms [22–24]. TF-CBT consists of prolonged and/or narrative exposure through imaginal reliving with rescripting and cognitive restructuring [25]. EMDR consists of attending to memories and associations while simultaneously engaging in bilateral physical stimulation, such as eye movements, taps, or tones [26]. Research on CPTSD across all its domains in adults is limited due to the novelty of the formal diagnosis, with only two recent studies identifying prolonged exposure [27, 28] and EMDR [28] as effective for adults with CPTSD. Furthermore, there are a lack of studies from real-world clinical settings.

### Aims of study

We sought to evaluate the treatment model of a specialist inner-London CPTSD service and its effectiveness in patients with CPTSD. Our first aim was to identify whether the package of trauma-focused psychotherapy (TF-P) offered (TF-CBT, EMDR, or a TF-CBT plus EMDR) within the phased model approach was effective at reducing PTSD symptom severity in a real-world setting. Our secondary outcomes were the change in depressive symptoms, CPTSD using a proxy measure, and functional impairment. Further exploratory aims of this study were (a) to compare differences between groups receiving TF-CBT, EMDR, and TF-CBT plus EMDR and (b) to identify whether baseline clinical severity of PTSD and depressive symptoms influenced treatment response.

## Materials and Methods

### Treatment setting and process

The traumatic stress clinic (TSC) is a local outpatient service within the UK National Health Service. The service assesses and treats adult patients with multiple, severe traumas and PTSD, and other comorbid difficulties. The TSC has specialist expertise in working cross-culturally with refugees, asylum-seekers, torture, developmental trauma survivors, victims of trafficking, and complex presentations. Referral criteria include a primary PTSD or CPTSD diagnosis, and readiness to talk about past traumas in treatment without experiencing high levels of emotional dysregulation. The service is unable to accept patients who cannot tolerate TF-P, that is, with significant difficulties with self-harm, drug and alcohol dependence, or other harmful ways of responding to distress.

### Patient referrals and treatment

Treatment at the TSC follows a phase-based approach [18–20]. In phase 1, up to five sessions of stabilization occur individually or in a group, and include PTSD psychoeducation, grounding techniques for flashbacks and nightmares, and exercises to improve anxiety and sense of safety. Clinicians may signpost clients for practical problems, for example, regarding finances and housing. Subsequently, patients are placed on a waitlist for TF-P.

Phase 2 involves processing traumatic memories to re-appraise associated emotions and meanings and integrate them into adaptive representations of the self, relationships, and world. Three TF-P options are offered: TF-CBT, EMDR, and TF-CBT combined with EMDR. Choice of therapy was influenced by clinician availability, expertise, and patient preference. TF-CBT at the TSC also draws on evidence-based treatments for multiple and complex traumas, such as narrative exposure therapy [29] and compassion-focused therapy [30]. Depending on clinical presentation, some patients are invited to attend a compassion-focused therapy group before, during, or after individual therapy [30, 31]. Unfortunately, we had insufficient information to incorporate this in our analysis. Phase 3, re-integration, builds on the hopes and goals of patients during treatment, encouraging the re-establishment of social and cultural connection. While we did not study this treatment phase, re-integration begins to be considered during phase 2 TF-P.

### Participants and procedures

Our sample included all TSC discharges between July 2016 and June 2019, satisfying the “selection” criterion in the assessment of methodological quality of case reports [32]. Eligible patients were all adults, had sustained multiple and prolonged traumata and had completed outcome measures at assessment, start of treatment, and end of treatment. Using a pseudonymized list of yearly discharges, we classified patients as meeting ICD-11 diagnostic criteria for CPTSD retrospectively through standardized psychological measures, file review and consultation with expert treating clinicians, fulfilling criteria for “ascertainment” in the evaluation of the methodological quality of case reports [32]. Patients had to meet CPTSD criteria across all three steps to be included in the study.

First, the presence of symptoms based on items of the Post-traumatic Checklist (PCL)-5 [33], Patient Health Questionnaire (PHQ)-9 [34], and Work and Social Adjustment Scale (WSAS) [35] corresponding to the ICD-11 diagnosis of CPTSD (see Table 1) were evaluated.

**Table 1.** Items used to assess for ICD-11 complex PTSD.

ICD-11 symptoms	PCL-5, PHQ-9, and WSAS items capturing CPTSD symptom clusters
Re-experiencing	PCL-2: Repeated, disturbing dreams of the stressful experience?
	PCL-3: Suddenly feeling or acting as if the stressful experience were actually happening again (as if you were actually back there reliving it)?
Avoidance	PCL-6: Avoiding memories, thoughts, or feelings related to the stressful experience?
	PCL-7: Avoiding external reminders of the stressful experience (e.g., people, places, conversations, activities, objects, or situations)?
Hyperarousal	PCL-17: Being “superalert” or watchful or on guard?
	PCL-18: Feeling jumpy or easily startled?
Affect dysregulation	PCL-14: Trouble experiencing positive feelings (e.g., being unable to feel happiness or have loving feelings for people close to you)?
	PCL-15: Irritable behavior, angry outbursts, or acting aggressively?
Negative self-perception	PCL-10: Blaming yourself or someone else for the stressful experience or what happened after it?
	PHQ-6: Feeling bad about yourself or that you are a failure or have let yourself or your family down
Interpersonal problems	PCL-13: Distant and cut-off from people
	WSAS-5: Because of my [problem], my ability to form and maintain close relationships with others, including those I live with, is impaired.

*Note*. A score of >2 was required for a symptom to be considered endorsed for the PCL-5 and PHQ-9, and a score of >4 for the WSAS.

*Abbreviations:* CPTSD, complex post-traumatic stress disorder; ICD-11, International Classification of Diseases; PCL, Post-traumatic Checklist; PHQ, Patient Health Questionnaire; WSAS, Work and Social Adjustment Scale.

The second step involved reviewing clinical case notes to confirm that the patient fulfilled all CPTSD domains. Affect dysregulation was endorsed when clinicians described emotional reactivity, dissociation, high levels of anger, aggression, and/or emotional numbing [36]. Negative self-concept was operationally defined as persistent negative beliefs about the self, and feelings of guilt and shame related to the event. Interpersonal disturbances included social isolation, avoidance of family, friends, intimate relationships; estrangement; and difficulty with emotional intimacy [36].

In the third step, we consulted clinicians involved in patients’ care to ascertain whether patients fulfilled the criteria for CPTSD at assessment. Clinicians were blind to the rating derived from clinical notes and questionnaires, and reported whether each ICD-11 CPTSD symptom was present.

### Measurements

Sociodemographic characteristics and The Life Events Checklist (LEC) [37] were collected at baseline. Outcome measurements were collected at assessment, start of treatment, and end of treatment.

### PTSD symptoms

The PCL-5 [33] is a 20-item self-report measurement of PTSD based on the DSM-5 [38]. Scores range from 0 to 80 and refer to the past month. A 10-point reduction represents clinically significant change, and a cut-off of 33 indicates a PTSD diagnosis [39]. It has been reported to have good psychometric properties [40].

### Depressive symptoms and functional impairment

The PHQ-9 [34] is a self-report instrument measuring nine DSM-IV [41] criteria for depression. Scores range from 0 to 27, with higher scores reflecting depression severity. It is well-validated [34] with good sensitivity to change [42]. A five-point reduction on the PHQ-9 [43] reflects clinically significant change and a score of less than 5 reflects loss of diagnosis [44].

The WSAS is a five-item self-report rating scale, measuring perceived impairment in functioning in the domains of work, home management, social leisure activities, private leisure activities, and relationships with others. A WSAS score above 20 suggests at least moderately severe impairment from psychopathology [35].

### Proxy for the International Trauma Questionnaire (ITQ) to measure CPTSD

We calculated total scores for items used to screen for CPTSD, mapping onto symptom dimensions of CPTSD based on the ICD-11 and the ITQ [45] (see Table 1). PHQ-9 and WSAS item responses were converted to a five-item scale comparable to the ITQ and PCL-5.

### Adverse and no treatment effects

We recorded hospitalizations, suicide attempts, serious self-harm resulting in presentation to hospital, or severe deterioration in functioning and symptomatology due to treatment as documented in clinical notes. Symptom deterioration was measured through reliable change on the PCL-5 and PHQ-9 using the reliable change index (RCI) (see below).

### Statistical analysis

Linear multilevel mixed-effects models examined treatment effects on outcomes over time. The random component included a random subject intercept term to account for correlations between repeated measurements [46]. Fixed effects included: age, sex, the dummy variable of treatment period (assessment, start of treatment, and end of treatment), treatment time, and number of sessions. The fixed effects assessing change in the PTSD scores included baseline depression scores, treatment period, and depression interaction. The exploratory models assessing change in depression scores included baseline PTSD scores and their interaction with treatment period, and the model assessing change in functional impairment included baseline PTSD and depression scores. To explore clinical change in the treatment phases, we compared symptom change during stabilization and waiting versus during individual TF-P (i.e., pre-to-post phase 1 symptom change versus pre-to-post phase 2 symptom change) on primary and secondary outcomes using paired samples *t*-tests. Rates of reliable change [47] were calculated for all outcomes in both treatment phases. For each outcome, the standard error of measurement (*SE*_meas_) was calculated using the scale’s Cronbach’s alpha and the standard deviation of a normative sample. Subsequently, the pre-treatment and post-treatment differences were divided by the standard error of the difference (*S*_diff_), with the absolute value reflecting the RCI. A change index score of over 1.96 was considered reliable [47]. Independent samples *t*-tests were used to assess for differences between treatment groups, for each outcome of interest across timepoints. Analyses were conducted using IBM SPSS Statistics 22 and STATA v16.1 MP 4.

## Results

**Figure 1.**
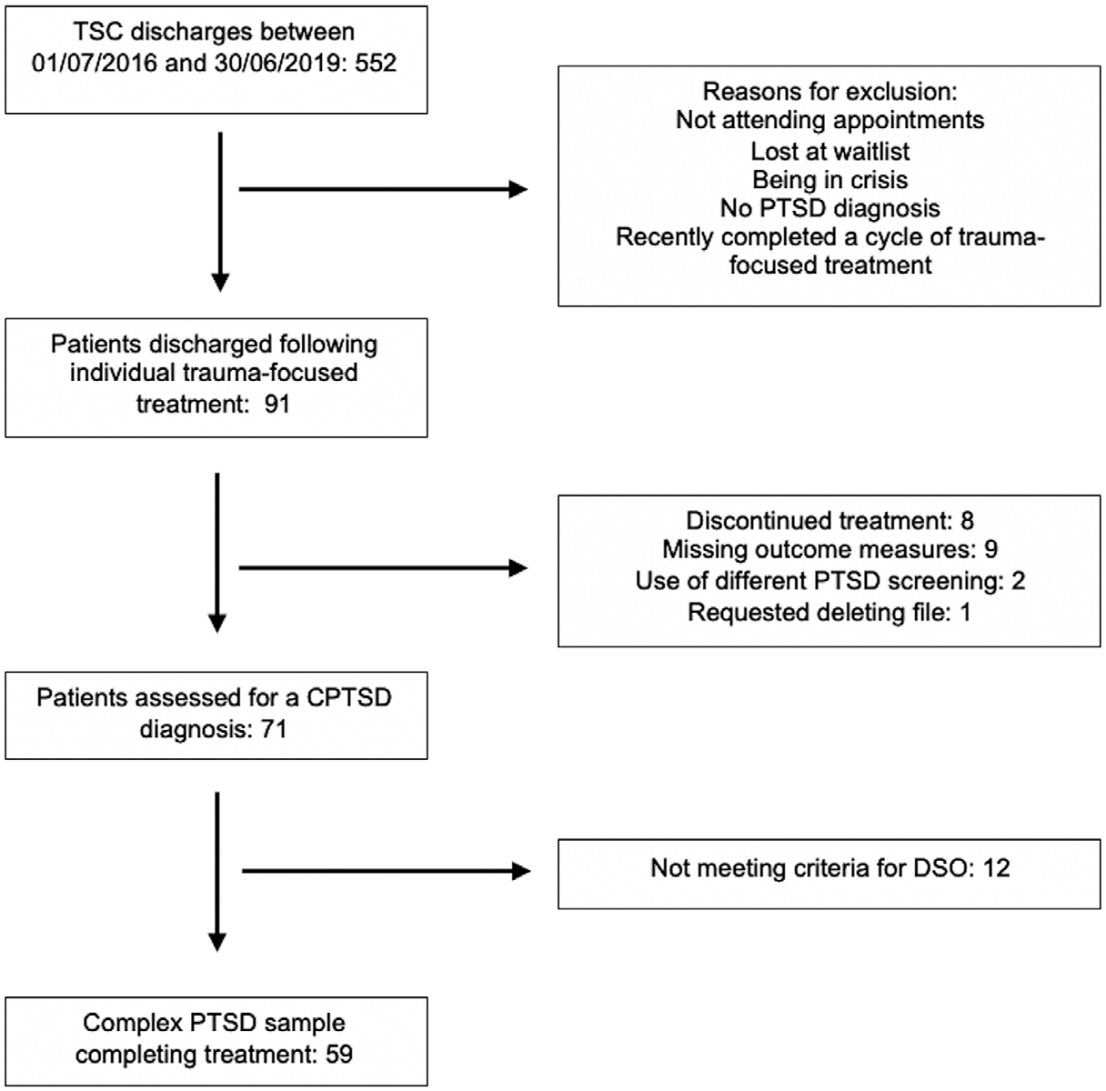
Flow diagram of participant classification with a complex post-traumatic stress disorder (CPTSD) diagnosis (DSO, disturbances of self-organization).

Figure 1 presents the screening of patients and reasons for exclusion, with 59 patients included in the study. Socio-demographic and clinical characteristics are presented in Table 2. Patients were aged between 25 and 63 years old [mean (*SD*) = 45.66 (9.19)] and 64% (*n* = 38) of them were female. Most patients reported psychiatric comorbidity (54.24%, *n* = 32) and received psychotropic medication (69.49%, *n* = 41). Most patients experienced developmental trauma and multiple traumatic events. Moreover, 84% endorsed directly experiencing at least three traumatic events on the LEC, with a mean of 5.09 (*SD* = 3.07) events directly experience. The sample was ethnically diverse, and 49.15% (*n* = 29) were of non-UK origin. whereas 35.60% (*n* = 21) were refugees or asylum seekers.

**Table 2.** Sociodemographic and clinical patient characteristics.

Age (years)	Mean (*SD*)		
	45.66 (9.19)		
	*n* (%)		*n* (%)
Sex^a^			
Male	21 (35.60)	Female	38 (64.40)
Ethnicity^b^			
White-British	28 (47.46)	Black-Caribbean	2 (3.39)
White-Other	7 (11.86)	Black-African	9 (15.25)
White-Irish	1 (1.70)	Other ethnic background	11 (18.64)
Asian-British	1 (1.70)		
Geographical region of origin^a^				
Northwestern Europe	31 (52.54)	North Africa	2 (3.39)
Southern Europe	1 (1.70)	Sub-Saharan Africa	7 (11.86)
Eastern European	6 (10.17)	Middle East	11 (18.64)
Asian	1 (1.70)		
Psychiatric comorbidity^a^			
Depression	24 (40.68)	Emotionally unstable personality disorder	2 (3.39)
Psychosis	3 (5.09)	Anxiety disorder	3 (5.09)
Type of index trauma^a^			
Developmental trauma	37 (62.71)	Domestic violence	14 (23.73)
Childhood emotional abuse	21 (35.59)	Traumatic bereavement	11 (18.64)
Childhood physical abuse	23 (38.98)	Torture	11 (18.64)
Childhood sexual abuse	25 (42.37)	Trafficking	3 (5.09)
Childhood neglect	7 (11.86)	Female genital mutilation	2 (3.39)
Childhood bullying	1 (1.70)		
Frequency of traumatic events (life events checklist)	*n* (%)		
Natural disaster	7 (11.86)	Unwanted sexual experience	23 (39.00)
Fire/explosion	4 (6.78)	War trauma/combat	13 (22.03)
Transportation accident	15 (25.42)	Captivity	18 (30.51)
Serious accident	10 (16.95)	Life threatening illness/injury	12 (20.34)
Exposure to toxic substance	8 (13.56)	Severe human suffering	14 (23.73)
Physical assault	34 (57.63)	Sudden violent death	4 (6.78)
Assault with a weapon	18 (30.51)	Sudden accidental death	17 (28.81)
Sexual assault	27 (45.76)	Serious injury/harm to others	2 (3.39)
Other stressful event or experience	19 (32.20)		
Number of medicines			
1	29 (49.15)	3	0
2	11 (18.64)	4	1 (1.70)
Psychopharmacological class (neuroscience-based nomenclature)^a^			
Serotonin reuptake inhibitor	21 (35.59)	Serotonin, norepinephrine-multimodal action	5 (8.47)
Serotonin, norepinephrine-reuptake inhibitor	3 (5.09)	Norepinephrine, serotonin-receptor antagonist (NE alpha-2, 5-HT2, 5-HT3)	13 (22.03)
Dopamine, serotonin-receptor antagonist (D2, 5-HT2)	1 (1.70)	Glutamate—Alpha-2 delta calcium channel blocker	3 (5.09)
Dopamine, serotonin-receptor antagonist (D2, 5-HT2), and reuptake inhibitor (NET) metabolite	3 (5.09)	GABA—Benzodiazepine receptor agonist (non-selective GABA-A receptor positive allosteric modulator)	1 (1.70)
GABA-PAM	4 (6.78)		

a Sex, geographical region of origin, psychiatric comorbidity, types of trauma, and information on medication were recorded based on each patient’s clinical case notes.

b Ethnicity categories were determined using the ethnic groups recommended for England and Wales, as described by the Office of National Statistics.

Mean phase 1 duration was 13.6 (8.1) months and TF-P duration was 17.60 (12) months. Mean (*SD*) number of phase 2 treatment sessions was 28 (10) (range: 7–60 sessions). In total, 57.60% (*n* = 34) received TF-CBT, 13.60% (*n* = 8) received EMDR, and 28.80% (*n* = 17) received TF-CBT plus EMDR. Outcome measurements by treatment group are presented in Table 3 and Table 4.

**Table 3.** Means, standard deviations, median, and maximum scores across TF-CBT, EMDR, and TF-CBT plus EMDR treatment groups.

Measure	TF-CBT	EMDR	TF-CBT plus EMDR
PCL-5 assessment [mean (*SD*)]	61.21 (11.56)	58.88 (11.14)	57.18 (11.25)
md (min–max)	62.00 (37.00–79.00)	55.50 (44.00–75.00)	59.00 (40.00–78.00)
PCL-5 start of TF-P [mean (*SD*)]	57.35 (12.19)	51.88 (18.07)	56.47 (13.16)
md (min–max)	59.00 (16.00–75.00)	50.50 (18.00–75.00)	54.00 (31.00–75.00)
PCL-5 end of TF-P [mean (*SD*)]	42.12 (16.06)	41.38 (22.98)	41.77 (18.42)
md (min–max)	47.00 (6.00–66.00)	42.50 (13.00–76.00)	37.00 (10.00–74.00)
PHQ-9 assessment [mean (*SD*)]	20.47 (4.15)	16.63 (6.63)	20.00 (4.14)
md (min–max)	21.00 (11.00–27.00)	14.00 (10.00–26.00)	21.00 (11.00–27.00)
PHQ-9 start of TF-P [mean (*SD*)]	19.06 (4.05)	17.88 (5.87)	20.29 (4.17)
md (min–max)	19.00 (11.00–27.00)	18.00 (10.00–26.00)	20.00 (13.00–27.00)
PHQ-9 end of TF- [mean (*SD*)]	14.16 (5.23)	13.25 (7.89)	15.35 (7.19)
md (min–max)	14.00 (02.00–25.00)	12.50 (2.00–27.00)	17.00 (4.00–27.00)
WSAS assessment [mean (*SD*)]	29.52 (6.99)	27.38 (5.81)	25.35 (9.10)
md (min–max)	30.00 (17.00–40.00)	25.50 (21.00–36.00)	23.00 (9.00–40.00)
WSAS start of TF-P [mean (*SD*)]	28.50 (5.62)	27.50 (6.74)	25.59 (8.60)
md (min–max)	30.00 (18.00–37.00)	25.50 (21.00–40.00)	24.00 (7.00–38.00)
WSAS end of TF-P [mean (*SD*)]	23.19 (8.36)	19.38 (11.38)	21.94 (13.10)
md (min–max)	24.00 (4.00–36.00)	19.00 (2.00–36.00)	16.00 (2.00–40.00)

*Abbreviations:* EMDR, eye movement desensitization and reprocessing; PCL, Post-traumatic Checklist; TF-CBT, trauma-focused cognitive behavioral therapy; TF-P, trauma-focused psychotherapy.

**Table 4.** Means and standard deviations across measurement points, and frequencies of clinical status at end of trauma-focused psychotherapy (TF-P).

	Assessment	Start of TF-P	End of TF-P	Clinically significant improvement at the end of TF-P	No longer meeting caseness at the end of TF-P
Measure	Mean (*SD*)	Mean (*SD*)	Mean (*SD*)	*n* (%)	*n* (%)
PCL-5	59. 73 (11.37)	56.36 (13.23)	41.92 (17.44)	32 (54.24)	20 (33.90)
PHQ-9	19.81 (4.64)	19.25 (4.35)	14.39 (6.18)	29 (49.15)	4 (6.80)
WSAS	28.00 (7.63)	27.49 (6.78)	22.28 (10.28)		

*Abbreviations:* PCL, Post-traumatic Checklist; PHQ, Patient Health Questionnaire; WSAS, Work and Social Adjustment Scale.

### PTSD symptoms

**Figure 2.**
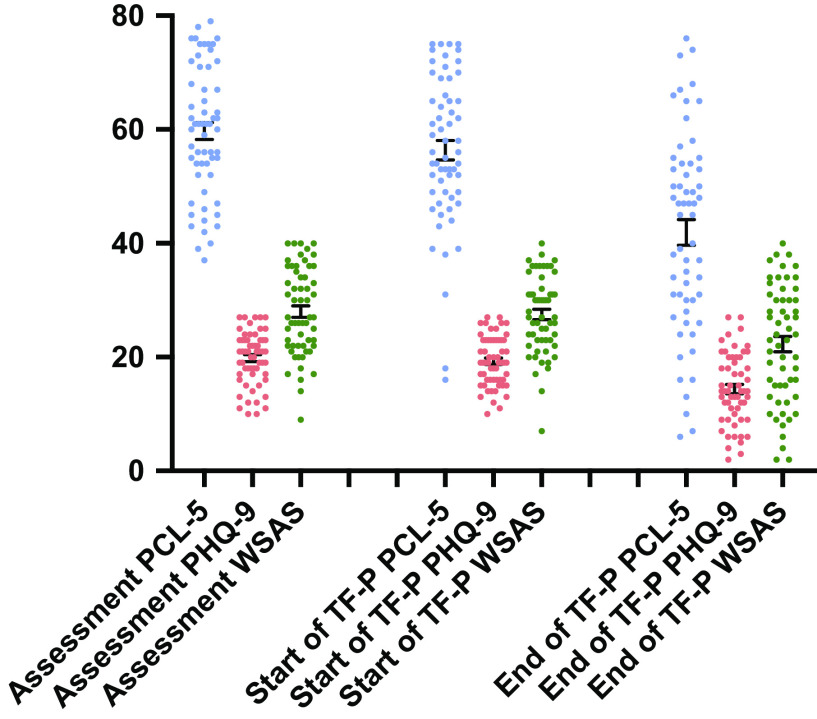
Individual post-traumatic stress disorder (PTSD checklist [PCL-5]) and depressive (Patient Health Questionnaire [PHQ-9]) symptom severity and psychosocial functioning (Work and Social Adjustment Scale [WSAS]) scores across measurement points. Error bars indicate standard error of measurement. TF-P, trauma-focused psychotherapy.

Patient outcomes across time are presented in Table 4. PCL-5 scores significantly improved following TF-P (coefficient −14.44; 95% CI −25.89 to −10.16) (see Table 2), with a large effect size (Cohen’s *d* = 0.89). PCL-5 scores did not significantly change during phase 1 (*p* = 0.162). Change in PCL-5 scores was significantly greater during TF-P [mean (*SD*) = −14.44 (16.21)] versus during phase 1 [mean (*SD*) = 3.37 (11.35)], *t*(58) = −3.99, *p* < 0.001 (Cohen’*s d* = 0.52) (see Figure 2). In total, 28.81% (*n* = 17) demonstrated positive reliable change during phase 1 and 54.24% (*n* = 32) demonstrated positive reliable change during phase 2. In total, 54.24% (*n* = 32) showed clinically significant change on the PCL-5 during phase 2 (see Table 2). Visually inspecting changes across domains of the PCL-5 showed a consistent reduction.

Baseline depression significantly and positively affected PCL-5 scores (coefficient 0.97; 95% CI 0.41 to 1.54) at the 5% level. There was no treatment period and baseline depression interaction (*p* > 0.49). No differences were observed in PCL-5 scores between patients receiving TF-CBT, EMDR, and TF-CBT plus EMDR, at any measurement point (all *p* > 0.42). There was no association between sex, age, number of sessions, time, and PCL-5 scores (all *p* > 0.57).

### Depressive symptoms, functional impairment, and CPTSD

The PHQ-9 had good internal reliability (*a* = 0.81). PHQ-9 scores significantly reduced following TF-P (coefficient −5.38; 95% CI −7.50 to −3.25) with a large effect size (Cohen’s *d* = 0.96). PHQ-9 scores did not significantly change during phase 1 (*p* = 0.51). Change in PHQ-9 scores during TF-P [mean (*SD*) = −5.07 (5.47)] was significantly greater than during phase 1 [mean *(SD*) = 0.56 (5.17)], *t*(58) = −4.41, *p* < 0.001 (Cohen’s *d* = 0.57) (see Figure 2). In total, 18.64% (*n* = 11) demonstrated positive reliable change during phase 1 and 40.68% (*n* = 24) demonstrated positive reliable change during phase 2. In total, 49.15% (*n* = 29) of patients showed clinically significant change on the PHQ-9 during phase 2.

Baseline PCL-5 score had a significantly positive effect at the 5% level on PHQ-9 scores (coefficient 0.14; 95% CI 0.02 to 0.25). The effect of baseline PTSD scores was consistent across measurement points, presenting no interaction with treatment period (all *p* > 0.337). Sex, age, number of sessions, or time was not associated with PHQ-9 scores (all *p* > 0.433). PHQ-9 scores did not differ between patients receiving TF-CBT, EMDR, or TF-CBT plus EMDR, at any measurement point (all *p* > 0.105).

The WSAS showed good internal reliability (*a* = 0.80). WSAS scores significantly decreased following TF-P (coefficient −5.11; 95% CI −8.52 to −1.71) with a moderate effect size (Cohen’s *d* = 0.54). WSAS scores did not significantly change during phase 1 (*p* = 0.580). Change in WSAS scores was significantly greater following treatment [mean (*SD*) = −5.21 (9.49)] than following phase 1 [mean (*SD*) = 0.33 (5.63)], *t*(58) = −2.26, *p* = 0.028 (Cohen’s *d =* 0.424) (see Figure 2). In total, 7.01% (*n* = 4) demonstrated positive reliable change during phase 1 and 34.48% (*n* = 20) demonstrated positive reliable change during phase 2. PHQ-9 (coefficient 0.51; 95% CI 0.06 to 0.97), but not PTSD (*p* = 0.195), scores had a significant effect on WSAS scores.

Sex, age, number of sessions, or time were not associated with WSAS scores (all *p >* 0.170). WSAS scores did not differ between patients receiving TF-CBT, EMDR, and TF-CBT plus EMDR, at any timepoint (all *p* > 0.185). In total, 59.3% (*n* = 35) continued to experience at least moderately severe impairment from psychopathology at the end of treatment.

There was no significant reduction in CPTSD severity during phase 1, *p* = 0.168. There was a significant reduction in CPTSD symptom severity from the start of treatment [mean (*SD*) = 34.49 (7.26)] to the end of treatment [mean (*SD*) = 25.47 (10.98)], *t*(58) = 7.18, *p* < 0.001 (Cohen’s *d* = 1.04). Change in CPTSD severity was significantly greater following treatment [mean (*SD*) = −9.05 (9.60)] than during phase 1 [mean (*SD*) = 1.36 (7.46)], *t*(58) = 4.69, *p* < 0.001.

### Adverse treatment effects

Regarding adverse effects, no hospitalizations, increased suicidality, or self-harm were reported to have occurred during treatment. Reliable worsening on the PCL-5 was observed in 11.86% (*n* = 7) of patients during phase 1 and 3.39% (*n* = 2) during phase 2. Reliable worsening on the PHQ-9 was observed in 8.48% (*n* = 5) of patients during phase 1 and 1.70% (*n* = 1) during phase 2. Reliable worsening on the WSAS was observed in 6.78% (*n* = 4) of patients during phase 1 and 3.39% (*n* = 2) during phase 2.

## Discussion

This is one of the first studies on the effectiveness of TF-P in improving PTSD symptoms in patients with CPTSD based on the ICD-11 criteria in a real-world setting. Depression, functional impairment, and CPTSD also improved significantly after treatment. Interestingly, higher depression scores were predictive of higher PTSD and impaired functioning across timepoints, and a smaller association was established with baseline PTSD and depression scores across timepoints.

### PTSD, depressive, and CPTSD symptoms

Positive reliable and clinically significant changes during TF-P were observed in more than half the sample. Comparing this to phase 1, where a third of patients reliably improved on PTSD symptoms, we see that in most patients PTSD symptoms do not tend to spontaneously improve over time in the absence of active TF-P. As we compared treatment with stabilization plus waiting, we cannot infer whether stabilization alone is effective. In two recent studies [27, 48], patients with CPTSD did not benefit more from the addition of affective and interpersonal skills training to prolonged exposure [27] and EMDR [48]. However, earlier research [49] had found additional skills training to improve outcomes for women with more severe difficulties in emotion regulation. It is therefore necessary for future research to elucidate the relative benefit of using a phase-based approach [22]. Additionally, as the PCL-5 is based on the DSM-5 diagnosis of PTSD [33], improvements in the DSM-5 domain “Negative alterations in cognition and mood” [38] may reflect changes in DSO.

Depression scores decreased significantly more during TF-P than during phase 1, in line with previous meta-analyses [23]. Approximately, half of the patients exhibited clinically significant change and 40.68% exhibited reliable improvement following TF-P. TF-CBT uses cognitive restructuring to change negative thinking patterns about the self and the world, such as negative thinking biases and dysfunctional core beliefs [25] also relevant in depressive symptoms, which have developed because of severe, repeated, and often chronic traumatic experiences.

The role of baseline depression on the trajectory of PTSD and functioning scores is noteworthy, as patients with CPTSD are known to experience worse levels of depression [16] and comorbid depression can negatively affect CPTSD treatment outcome [50, 51]. Putative explanations involve the way negative schemata and shame can interfere with the re-processing of trauma memories [52], but also how reduced motivation and hopelessness could make elements of treatment difficult to engage with. Depressive symptoms can be targeted through a multimodal approach [15], and in stabilization, especially if they significantly increase the risk of harm to self [19].

The statistically significant improvement in our proxy CPTSD score during TF-P needs to be interpreted with caution, given the retrospective and non-validated measurement. Treatment groups did not differ on symptoms across timepoints, consistent with meta-analyses comparing the effectiveness of TF-CBT to EMDR on both PTSD and depression scores [22, 23]. No sociodemographic characteristics were associated with clinical outcomes across timepoints. Although females have higher risk of CPTSD in population studies [53], the multiple and diverse range of traumas, and comorbidities observed in our sample may explain the consistent symptom severity.

### Adverse effects

Most past studies fail to describe adverse effects [24], despite the risk of increased PTSD symptoms, particularly re-experiencing, following TF-P [54, 55]. No adverse effects were reported by clinicians, but a small number of patients experienced reliable worsening on PTSD, depression, or functional impairment during treatment. The exclusion of patients dropping out of treatment could introduce selection bias to this finding.

### Strengths and limitations

Our study is novel in evaluating treatment in a sample meeting ICD-11 CPTSD diagnostic criteria in a real-world clinical setting with an ethnically and culturally diverse civilian sample. Our research on treatment following multiple traumas highlights the greater level of need compared to studies on single event traumas, providing a valuable addition to the current trauma literature. Finally, in contrast to previous research [23, 24], we considered adverse effects.

Limitations include a retrospective design and the absence of a separate control group. Adding to this the length of waiting time and treatment we need to consider the possibility of spontaneous remission. Varying levels of detail in clinical notes may have limited the retrospective ability to capture the clinical nature of a symptom, for example, depressive symptoms versus the negative self-concept and world view as part of DSO. However, our stringent process of participant selection by triangulating evidence from different sources would have provided some protection against this, increasing the internal validity of our measurement. Treatment comparison results could be explained by unadjusted confounding variables, as there was no randomization, and the sample size was small. The non-random provision of treatment modality may have been influenced by clinician availability, expertise, and preference. Another limitation is that we only included treatment completers with all outcome measures and without follow-up.

### Clinical implications

A clear clinical implication from our study concerns treatment length. More than half of the patients still met clinical diagnosis criteria after an average of 28 sessions, which is almost three times the number suggested by NICE clinical guidelines for PTSD [19]. This finding demonstrates that it is critical for specific CPTSD guidelines to be developed. Clinically, this population may present with shame and lack of trust arising from interpersonal traumas and require longer periods of time for engagement and the formation of a *good enough* therapeutic relationship [19]. A recent study [27] supported the view that longer treatment is needed, as some patients with CPTSD continue to present with elevated symptoms after therapy. We need to adapt treatments and available resources to fit these higher levels of complexity and severity [56].

Finally, although we did not record current life events that could interfere with treatment, more functional impairment is observed in CPTSD than in PTSD [3, 11, 13, 15, 27, 57]. This includes socioeconomic, relational, and housing difficulties. Consistent with meta-analyses [57], our sample maintained high levels of functional impairment following treatment. It is therefore essential to move beyond the narrow measurement of symptomatic change, to promoting wellbeing in all life domains affected by the debilitating experience of CPTSD.

### Suggestions for future research

Further research should determine the comparative efficacy and optimal sequence of different treatments with randomized controlled trials, and designs to identify personal markers of treatment effectiveness for CPTSD. Psychotherapeutic approaches that can improve one’s attachment organization and adaptive self and interpersonal schemata should be explored [52], and for whom which TF-P is most appropriate and safe.

## Data Availability

The data that support the findings of this study are available from the corresponding author M.B. upon reasonable request.
